# This I believe: we need to understand evolution, adaptation, and phenotype

**DOI:** 10.3389/fgene.2012.00303

**Published:** 2013-01-02

**Authors:** Eugenie C. Scott

**Affiliations:** Executive Director, National Center for Science Education, Inc.Oakland, CA, USA

I believe that there are three genetics-related concepts that, if taught properly, would greatly improve the biological literacy of our fellow citizens.

The first is evolution, the big idea of biology, that living things have common ancestors. Misunderstandings about the concept of evolution could produce an entire new column, but suffice to say that this basic biological principle, explaining why phenomena in biology are the way they are, rather than some other way, somehow hasn't filtered into American scientific literacy.

It is genes that make evolution possible. All eukaryotic organisms are related in a massive tree of life that includes organisms familiar and unfamiliar, linked through the transmission of genes from generation to generation over the last 2 billion years. The differential transmission of genes over time produces evolutionary change, and ultimately, with speciation, the branching of the tree of life. In addition to being fundamental to biology, this realization of our linkage through genes to every other living thing on the planet, past and present, has profound religious and philosophical implications.

Genes produce traits that either do or do not suit an organism for the environment in which it finds itself. This brings us to the second basic concept, adaptation. Natural selection is *adaptive* differential reproduction. Some genes produce traits that enable an organism to survive better and reproduce more in a particular environment. Those traits and the genes that affect them will become more prevalent over time, as long as the environment favors them. Those same genes, in a different environment, might not be adaptive, and because environments change, “perfect” adaptation neither occurs nor is expected—but nobody said the concept of natural selection was simple. Natural selection is best understood as survival of the *fit enough*.

Natural selection was not immediately accepted when Charles Darwin proposed it in the nineteenth century: under the prevailing blending theory of heredity, there was no mechanism to provide for new variation each generation. That obstacle was removed by Mendelian (particulate) genetics, which today, supplemented with molecular genetics, provides the grounding for the modern understanding of natural selection, which allows a much finer-grained understanding of the fit of species and populations to their environments.

The implications of the modern, genetically-based theory of evolution by natural selection for our own species are particularly profound. All humans belong to a single species and share a common descent, there is great genetic diversity in all human populations, and no human group enjoys a monopoly on the traits that allow for humans to be a successful species. Nor, because of the way genes, adaptation and evolution operate, was it ever true that there were (or are) “master races” or even “superior races”: environments change, and what is advantageous at one time and place will not necessarily always be so. Evolution is thus a powerful scientific bulwark against racism (even though, admittedly, it has been misrepresented and misused in the service of racist agendas).

Genes interacting with the environment leads us to the third important concept for biological literacy: the phenotype. Properly understood, the phenotype is not the textbook simplification “what you see,” but the result of the effect of genes plus the environment plus their interaction (That last component is rather of more interest to specialists than the general public). Both genes and environment play a role: East Africa produces a disproportionate number of distance runners partly because of the small and lightweight bodies of its inhabitants, but also because of a culture that values running and regards competitive running as a ticket out of poverty.

Genetic determinism is thus false and should be avoided. Perhaps there is no need to stress that point, given the excesses of Nazism and other genocidal ideologies; “genetic determinism” is deservedly often used as an epithet. Genes and other biological factors produce organisms, but they do not determine them. The soundbite version: we may be 100% genetic, but we're not 100% determined.

But environmental or cultural determinism is also false and should also be avoided: even highly environmentally-influenced human traits, such as personality, sexual orientation, intelligence, aggression, and the like, still are phenotypes, with genetic as well as environmental components influencing their expression. Yes, the Tarahumara of the canyons of northwest Mexico value running to such a degree that they are famous for their 48-h jogs covering hundreds of miles. But recognizing the cultural forces at work here should not preclude asking the physiological question of whether the Tarahumara are genetically equipped to process energy more efficiently than the rest of us. If we are cultural determinists, we will never think to ask that question.

An understanding of the true meaning of the concept of phenotype would lead us to a better understanding of not only basic biology, but also prickly issues of race, sex, and behavior. Consider the recent election season, in which there were a fair number of women, African-Americans, Asian-Americans, and lesbian, gay, bisexual, and transgender candidates for office. How often did you encounter, explicitly or implicitly, the misguided idea that their genetically-based characteristics either qualified or disqualified them? Such incidents remind us how useful the concept of phenotype actually is. I repeat: we are 100% genetic—but not 100% determined.

Evolution, adaptation, and phenotype. If teachers could do a better job teaching these concepts, Americans would be more biologically literate, which—dare I hope?—might lead to more thoughtful conclusions about what it means to be human.

